# Hierarchical Ni(OH)_2_/Cu(OH)_2_ interwoven nanosheets *in situ* grown on Ni–Cu–P alloy plated cotton fabric for flexible high-performance energy storage†

**DOI:** 10.1039/d0na00210k

**Published:** 2020-06-05

**Authors:** Man Zhou, Zhihang Jin, Lifang Su, Kai Li, Hong Zhao, Jinguang Hu, Zaisheng Cai, Yaping Zhao

**Affiliations:** Key Laboratory of Science and Technology of Eco-Textiles, Ministry of Education, College of Chemistry, Chemical Engineering and Biotechnology, Donghua University Shanghai 201620 P. R. China zhaoyping@dhu.edu.cn; Fundamental Experimental Chemistry Center, Donghua University Shanghai 201620 P. R. China; Department of Chemical & Petroleum Engineering, Schulich School of Engineering, University of Calgary Calgary AB T2N 4V8 Canada

## Abstract

Flexible energy storage electrodes with high conductivity and capacity are crucial for wearable electronic clothes. Herein, a flexible hierarchical Ni(OH)_2_/Cu(OH)_2_ interwoven nanosheets *in situ* coated on Ni–Cu–P alloy plated cotton fabric textile (NCO/CF), which displays perfect conductive and electrochemical performance, is prepared by electroless deposition and electrochemical oxidation method. While the Ni–Cu–P alloy layer coated on the fabric effectively contributes to excellent mechanical performance and electro-conductivity of the as-prepared NCO/CF electrode, the hierarchical Ni(OH)_2_/Cu(OH)_2_ interwoven nanosheets in the oxidation layer effectively lead to a high energy storage performance with a specific areal capacity of 4.7 C cm^−2^ at a current density of 2 mA cm^−2^. When the power density of the two-electrode system based on NCO/CF and the carbon cloth (CC) is 2.4 mW cm^−2^, the energy density is 1.38 mW h cm^−2^. Furthermore, the flexible solid-state energy storage f-NCO/CF//CC is assembled in a self-powered system and supplies continuous power for electronic devices, demonstrating that NCO/CF is promising to be applied in various energy storage devices to power portable and wearable devices in the future.

## Abstract 2

Flexible Ni(OH)_2_/Cu(OH)_2_@Ni–Cu–P alloy coated on cotton fabric with high conductivity and excellent mechanical performance is available for future smart and wearable electronic devices.

## Graphical abstract

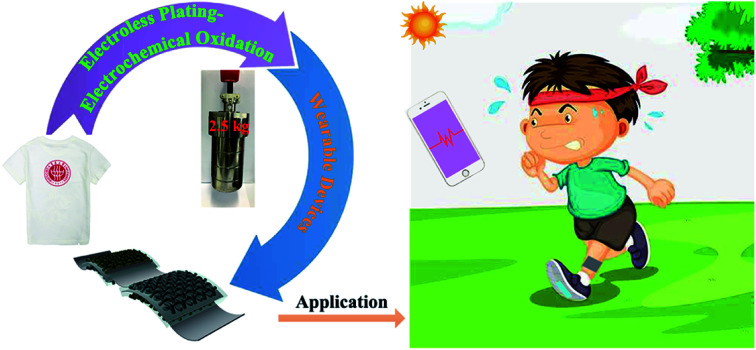

## Introduction

With the increase in demand for portable devices, roll-up electronics, and smart textile for sports activities and healthcare, the flexibility of energy conversion and storage has become a focus for developing novel techniques.^1–6^ Because textile is a necessity for human beings in daily life, wearable energy storage devices (ESDs), including supercapacitors, batteries and hybrid devices, based on yarns and fabrics is a popular trend. To date, multiple fabrication methods for textile-based ESDs have been reported, including carbonization, printing, dip-coating and *in situ* chemical growing electroactive materials.^7–11^ When fabricating a textile-based ESD, electroconductivity is the necessary prerequisite for high power density. Graphene, carbon nanotubes (CNTs), conducting polymers, and metallic nanoparticles are mostly used to modify the conductivity of textile substrates.^8–13^ As an alternative, electroless plating has been a promising method to obtain a metal-coated textile-based current collector due to the excellent conductivity of metals.^12,14,15^ Compared with metal foils, foams or meshes, electroless plated textiles as a current collector can not only realize flexibility but also additional loading amount because of their high surface area.

Because of excellent uniform plating ability, good abrasion resistance and corrosion resistance, electroless nickel–phosphorus (Ni–P) plating is extensively applied in the fields of petrochemicals, industrial machinery, aerospace, and electrical and electronic applications.^15,16^ The chemical deposition of multiple alloys is an effective method to further improve the properties of Ni–P alloys. Note that the Ni–Cu–P alloy has been reported in different fields with both high electric conductivity and low corrosion.^17–19^

Among various electrochemically active materials, Ni-based oxides and hydroxides are considered as promising candidates owing to low cost, good electrochemical performance and environmental friendliness. Moreover, they have a high theoretical specific capacity of 1292 C g^−1^ and 1041 C g^−1^ for NiO and Ni(OH)_2_, respectively.^20,21^ Cu-based hydroxides have attracted considerable interest because they have various microstructures and abundant reaction sites.^22–25^ However, their electrochemical performance is limited by poor electroconductivity.^20,26,27^ It is reported that Ni- and Cu-(hydro)oxide hybrids have better conductivity compared with that of single-phase Ni or Cu due to the increased oxygen vacancies from lattice defects.^28,29^

In this work, a hierarchical Ni–Cu hydroxide interwoven nanosheets *in situ* grown on Ni–Cu alloy plated cotton fabric (CF) was fabricated by a top-down strategy through Pd-free electroless plating, followed by *in situ* electrochemical oxidation. In detail, the CF was metalized with Ni–Cu–P alloy by Pd-free electroless plating. As a textile-based flexible current collector, the coated Ni–Cu–P alloy acted as the precursor of Ni- and Cu-based hydroxides through *in situ* electrochemical oxidation. To our knowledge, this is the first time that Ni–Cu–P alloy metalized cotton fabric was used as the current collector for energy storage. Compared with traditional methods of electrode fabrication, using insulative and inactive binders or growing active materials on the current collectors, our method realizes an intrinsically integrated active flexible electrode material using a simple fabrication process. This design gives rise to a robust combination between the active materials and Ni–Cu–P alloy plated cotton fabric substrate, which indicates a strong electronic contact and good conductivity. Furthermore, the hierarchical interwoven nanosheet structure provides less diffusion resistance for electrolyte ions and a high specific surface area with adequate active sites for energy storage. Therefore, the as-prepared NCO/CF delivers excellent electrochemical performance. At the current density of 2 mA cm^−2^, the specific areal capacity is as high as 4.7 C cm^−2^.

## Results and discussion

### 2.1 Synthesis method of the NCO/CF flexible electrode

**Fig. 1:**
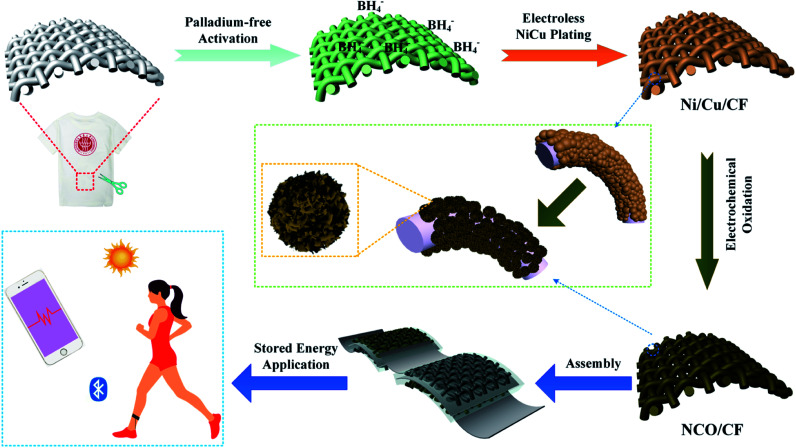
Schematic of the fabrication process of NCO/CF.

The schematic of the fabrication process of NCO/CF is presented in Fig. 1. The Ni–Cu–P alloy plated CF decorated by Ni(OH)_2_/Cu(OH)_2_ hybrid was fabricated through facile electroless plating and electrochemical oxidation method. First, the clean CF was plated with the electroless plating process in the plating bath using three concentration ratios of Cu to Ni salt in the electroless plating bath (1 : 12, 1 : 9 and 1 : 6). The plated samples with Ni–Cu–P were denoted as Ni/Cu/CF-1, Ni/Cu/CF-2, and Ni/Cu/CF-3. Followed by the electrochemical oxidation on the surface of the plated samples, the as-prepared electrode samples were NCO/CF-1, NCO/CF-2, and NCO/CF-3.

### 2.2 Morphology and chemical composition of the NCO/CF flexible electrode

**Fig. 2:**
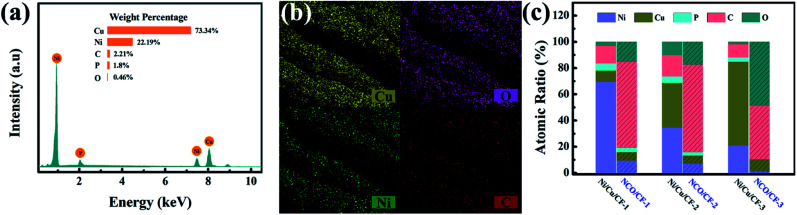
(a) EDS of Ni/Cu/CF-3. (b) Elemental mappings of NCO/CF-3. (c) Bar graph for composition content of Ni/Cu/CFs and NCO/CFs coating.

The elemental composition of the Ni–Cu–P alloy plated CF samples Ni/Cu/CF-1, Ni/Cu/CF-2 and Ni/Cu/CF-3 (Ni/Cu/CFs) was explored by EDS. Represented typically by Ni/Cu/CF-3, as shown in Fig. 2a, Ni/Cu/CFs primarily have Ni, Cu, P, C and O elements, in which Ni, Cu, and P are from the Ni–Cu–P alloy deposits and C and O are mainly derived from the CF substrate. As shown in the part without the shadow area in the bar graph in Fig. 2c, the atomic ratios of Cu in Ni–Cu–P deposits significantly increase as the concentration of Cu salt in electroless plating bath increases while the Ni and P content present in these coatings decreases. This behavior can be explained by the fact that Cu is preferentially deposited because the reduction potential of Cu is higher than that of Ni.^30,31^ The preferentially precipitated Cu increases the proportion of non-catalytically active parts on the surface, which suppresses Ni reduction. Then, the reduction of catalytically active Ni content in the coating causes the reduction of P content.

After electrochemical oxidation or electro-corrosion, the atomic ratios for Cu, Ni, O, and C in three NCO/CFs from EDS are shown in the part with the shadow area in the bar graph in Fig. 2c. Note that P element content decreases in NCO/CFs compared to that in Ni/Cu/CFs. In particular, in NCO/CF-3, the P element cannot be even detected from EDS. Combined the decrease of P element with the increase of O element, they can be an indication of the extent of the electro-erosion. Accordingly, NCO/CF-3 is the product of the most easy electro-corrosion from Ni/Cu/CF-3, which has the lowest P content. This is in accordance with the reduced P content, which leads to poorer corrosion resistance.^32^ Moreover, the SEM-EDXS elemental mapping in Fig. 2b shows that Cu, Ni, O and C are homogeneously distributed on the surface of the fibres in NCO/CF-3.

**Fig. 3:**
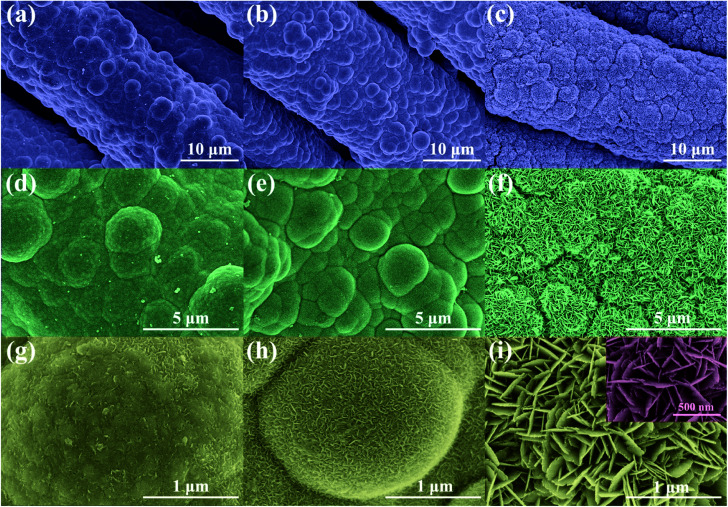
FESEM images of NCO/CF-1 (a, d and g), NCO/CF-2 (b, e and h) and NCO/CF-3 (c, f and i) with different magnifications.

The morphology of Ni/Cu/CF and NCO/CF samples was examined and shown in Fig. 3 and S1.† According to Fig. S1a and b,† CF is uniformly coated by Ni–Cu–P alloy after electroless plating. Note that Ni/Cu/CF-3 in Fig. S1c† shows Ni–Cu–P particles with a rugged surface on the cotton fibre after electrochemical oxidation. Furthermore, as shown in Fig. 3f and i, NCO/CF-3 has a more distinguishable hierarchical nanostructure under high magnification. Interestingly, there exists considerably high content of O from EDS in NCO/CF-3 as shown in the part without the shadow area in the bar graph in Fig. 2c compared to the other two samples. This illustrates that more oxide or hydroxide is formed in NCO/CF-3 along with denser nanostructures as shown in Fig. 3c, f and i, compared with that for NCO/CF-1 in Fig. 3a, d and g and NCO/CF-2 in Fig. 3b, e and h. In detail, with increase in the ratio of Cu salt in the electroless plating bath, the obtained NCO/CFs show more obvious nanosheet-like structure on the surface. While NCO/CF-1 only shows an embryonic form (Fig. 3g), some nanowire structures can already be observed (Fig. 3h) on NCO/CF-2. Further, for NCO/CF-3, the surface is covered with densely interwoven nanosheets (Fig. 3i). These nanosheets have *ca.* 200–300 nm of width and *ca.* 15–20 nm of thickness, which interlaces with each other and forms a large number of macropores and mesopores, as shown in the inset of Fig. 3i. This nanostructure considerably increases the specific surface area of the materials, which can reduce the distance of ion transport and optimize kinetic properties during energy storage.

**Fig. 4:**
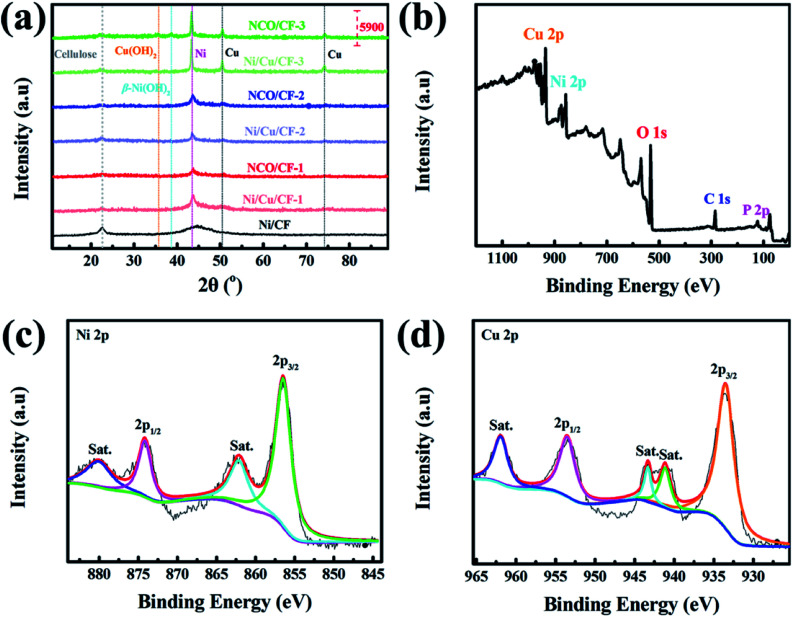
(a) XRD patterns of CF, Ni/CF, Ni/Cu/CFs and NCO/CFs. (b) XPS survey spectrum, and high-resolution XPS spectra for (c) Ni 2p, and (d) Cu 2p of NCO/CF-3.

The crystalline phases of CF, Ni/CF, Ni/Cu/CFs and the corresponding samples after electrochemical oxidation, NCO/CFs, were investigated using X-ray powder diffraction (XRD). As shown in Fig. 4a, all samples show a peak at 2*θ* = 22.4°, which is ascribed to the (001) crystal plane of the cellulose from the CF substrate. In Ni–CF, there is a broad diffraction peak at 2*θ* = 40°–50° from amorphous Ni–P coating because of the absence of Cu. In comparison, as for the samples under lower copper content, Ni/Cu/CF-1 and NCO/CF-1, a broad peak with low intensity can be observed at 2*θ* = 44.2°, which corresponds to the (111) crystal planes of Ni phase and indicates low crystallinity. With the copper content gradually increasing in electroless plating baths, it can be clearly observed that peaks appear at 2*θ* = 44.2°, 50.3° and 74.1°, corresponding to the (111), (200) and (220) crystal planes, with higher intensity in XRD patterns of Ni/Cu–CF-2, Ni/Cu–CF-3, NCO/CF-2 and NCO/CF-3, respectively. These peaks are in accordance with the standard patterns of pure Ni (PDF No. 04-0850) or pure Cu (PDF No. 04-0836) with a face-centered cubic (fcc) structure. Obviously, with the Cu content increasing and P content decreasing, the microstructure of the Ni–Cu–P alloy layer gradually changes from amorphous to crystalline, which is agreement with previous reports.^33,34^ After electrochemical oxidation, compared with NCO/CF-1 and NCO/CF-2, only NCO/CF-3 shows the peak of Cu(OH)_2_ at 2*θ* = 35.5° and the peak of (101) crystal plane of β-Ni(OH)_2_ at 2*θ* = 38.6° changes, which is relative to the fact that Ni/Cu/CF-3 is easily electro-corroded with the lowest P content. These results are in accordance with the EDS and SEM results.^35,36^

The chemical composition of NCO/CFs was further explored by X-ray photoelectron spectroscopy (XPS). The survey spectrum and high-resolution XPS spectra are shown in Fig. 4b, c and S2,† respectively. Typically, the survey spectrum of the NCO/CF-3 primarily shows Cu, Ni, O, C and P species in Fig. 4b. The peaks located at 934.0, 856.0, 532.0, 285.0 and 76.0 eV are assigned to the characteristic peak of Cu 2p, Ni 2p, O 1s, C 1s and P 2p, respectively. In terms of the Ni 2p XPS spectrum in Fig. 4c, the Ni 2p spectrum can be best fitted by considering the spin–orbit doublet characteristic of Ni^2+^ at 856.5 and 874.1 eV, which is attributed to the binding energy of Ni 2p_3/2_ and Ni 2p_1/2_ with a spin-energy separation of 17.6 eV. This behavior is the characteristic of a Ni(OH)_2_ phase and in good agreement with previous studies.^37^ Furthermore, two peaks at 862.1 eV and 880.0 eV are the shake-up satellites of the major Ni 2p_3/2_ and Ni 2p_1/2_.^35,38^ As shown in Fig. 4d, the peak-fit of Cu 2p_3/2_ peaks displays a primary peak at 933.5 eV, which is accompanied by two satellite peaks at 941.1 and 943.3 eV, respectively. The binding energy positioned at 953.5 eV is derived from the binding energy Cu 2p_1/2_ with a satellite peak positioned at 961.9 eV. These features correspond to a Cu^2+^ state for Cu(OH)_2_.^35,39^

The specific surface area and the pore structure of the as-prepared NCO/CF samples were investigated by N_2_ adsorption–desorption isotherm and pore size distribution (PSD) based on the BJH method. As shown in Fig. S3a,† they all show a type IV isotherm with a H_3_-type hysteresis loop, illustrating the presence of mesopores. Brunauer–Emmett–Teller (BET) specific surface areas are 174.86, 270.97 and 319.57 m^2^ g^−1^ for NCO/CF-1, NCO/CF-2 and NCO/CF-3, respectively. The average pore diameter is in the mesoporous region with the main pore size of 2–3 nm, as shown in Fig. S3b.†

### 2.3 The electrochemical performance of the NCO/CF flexible electrode

To study the electrochemical properties of NCO/CFs as the energy storage electrodes, the cyclic voltammetry curves (CV) were first tested. The CV curves for NCO/CF-3 at various scan rates are shown in Fig. S4.† A pair of redox peaks can be visibly observed, which is ascribed to the battery-type electrochemical reactions of Ni(OH)_2_ and Cu(OH)_2_ in 2 M KOH as shown in the equation below.^21,35,40,41^Ni(OH)_2_ + OH^−^ ↔ NiOOH + H_2_O + e^−^2Cu(OH)_2_ + 2e^−^ ↔ 2CuOH + 2OH^−^ ↔ Cu_2_O + H_2_O + 2OH^−^

**Fig. 5:**
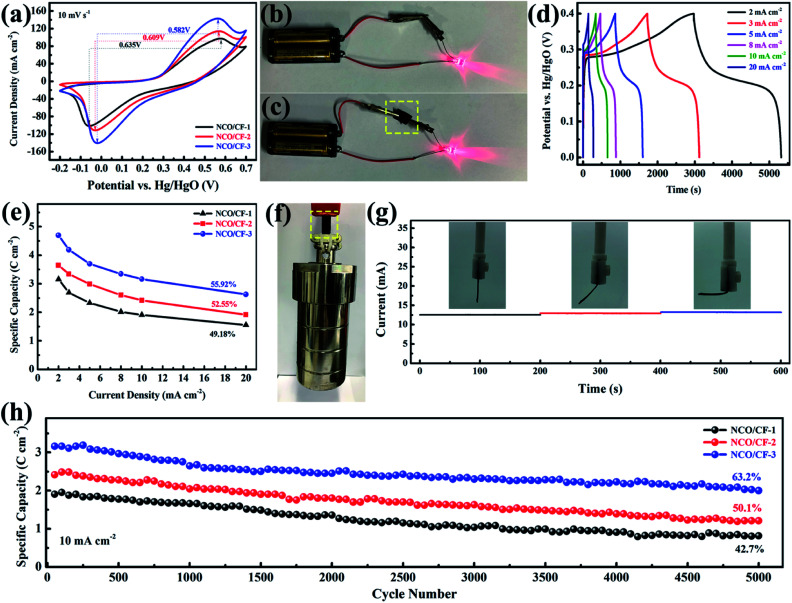
(a) CV curves of NCO/CFs at a scan rate of 10 mV s^−1^. (b and c) The optical pictures of NCO/CF-3 acted as a wire. (d) GCD curves of NCO/CF-3 at various current densities. (e) The specific areal capacity of NCO/CFs at various current densities. (f) The optical picture of NCO/CF-3 suspending a ∼2.5 kg of hydrothermal autoclave reactor. (g) Current–time curves of NCO/CF-3 bent with different states under a constant voltage. (h) The cyclic life test of NCO/CFs.

Note that as the scan rate increases, the redox potential shifts to higher and lower potentials because of polarization. Further, the CV curves for three NCO/CF samples at a scan rate of 10 mV s^−1^ are shown in Fig. 5a, where the spacing between the redox peaks decreases from 0.635 to 0.582 V for NCO/CFs with the Cu content in the substrate increasing. Moreover, the average voltage of the redox peaks for them gradually increases from 0.260 to 0.271 V. The spacing between the redox peaks of the reaction represents the reversibility of the reaction. The smaller the spacing, the better the reversibility. The average voltage of the redox peaks represents the reaction potential of the electrochemical reaction. An increase in the reaction potential allows the electrode to exchange additional energy under the same current conditions. NCO/CF-3 shows the smallest spacing and the largest average voltage for the redox peaks due to the best conductivity of the substrate from the highest Cu content. Therefore, the electrode can transmit the charge faster in the redox reaction, which eliminates the polarization to some extent, thus enhancing the reversibility and the reaction potential of the electrode reaction.^42^ The excellent conductivity of NCO/CF-3 can be confirmed by Fig. 5c, in which it is the equivalent of the role of wire, similar to the metal clips in Fig. 5b. In Fig. 5d, the galvanostatic charge–discharge curves (GCD) of NCO/CF-3 at various current densities are shown. Accordingly, the specific capacity is 4.7, 4.2, 3.7, 3.3, 3.2 and 2.6 C cm^−2^ at the current density of 2, 3, 5, 8, 10 and 20 mA cm^−2^, with increase in coulombic efficiency of 79.0%, 81.1%, 85.6%, 90.2%, 92.5% and 92.8%, respectively. This results from the side reactions along with the electrochemical redox reaction at low current density, which in turn leads to incomplete discharge. As shown in Fig. 5e, NCO/CF-3 displays the best energy storage performance among three NCO/CFs, with the highest specific capacity and the rate capacity, in accordance with the results from CV curves in Fig. 5a and GCD curves in Fig. S5.† Moreover, it can be further confirmed by the Nyquist plots from EIS analysis, as shown in Fig. S6.† The intercept of real impedance (Z′) at a high frequency range reflects a very low internal resistance of 1.56, 0.97 and 0.42 Ω for NCO/CF-1, NCO/CF-2 and NCO/CF-3, respectively, which is related to the inherent resistance of the active materials and the contact resistance.^43^ Moreover, the small diameter of the semicircle stands for the fast charge transport resistance. The slope of the curve tail at a low frequency range exhibits diffusion resistance. The steepest slope for NCO/CF-3 illustrates its reasonable diffusion limitation of the electrolyte,^3^ ascribed to its highest BET specific surface area and pore structure.

Based on the cotton fabric as the substrate, excellent mechanical strength and toughness of NCO/CF-3 electrode are expected. As shown in Fig. 5f, even when a hydrothermal autoclave reactor weighing up to 2.5 kg is suspended, NCO/CF-3 electrode can withstand without breakage fracture. Moreover, the current response of the electrode materials is not very different under different bending conditions in Fig. 5g, indicating that the electrode retains good flexibility while possessing better electrochemical performance. Such strength and toughness are unavailable for many self-supported flexible electrode materials. The superior flexibility and strength makes NCO/CF-3 more potential and competitive for the practical application of flexible energy storage devices in future. Moreover, the cyclability of NCO/CFs was evaluated based on 5000 times of galvanostatic charge–discharge tests at a current density of 10 mA cm^−2^. As shown in Fig. 5h, the NCO/CF-3 electrode capacity degradation is the least with 63.2% of the original capacity after 5000 cycles. This can be ascribed to its good electroconductivity. Compared to the original perfect nanosheets in Fig. 3c, f and i, the capacity degradation results from the collapse of the nanosheets as shown the NCO/CF-3 SEM images after 5000 GCD cycles in Fig. S7.† Furthermore, no obvious change for the XRD pattern in Fig. S8† compared with that before 5000 GCD cycles in Fig. 4a shows no crystal form transformation after cycling and exhibiting peaks for Cu(OH)_2_, β-Ni(OH)_2_, Ni and Cu.

### 2.4 The electrochemical performance of NCO/CF-3//CC two-electrode system

**Fig. 6:**
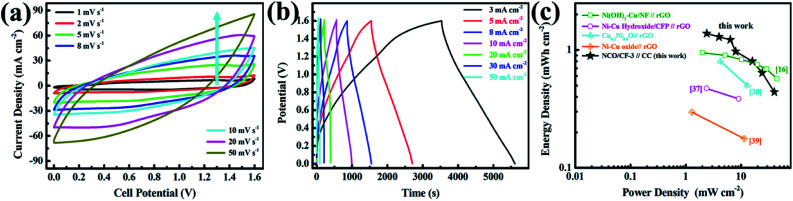
(a) CV curves of NCO/CF-3//CC at various scan rates. (b) GCD curves of NCO/CF-3//CC at various current densities. (c) Ragone plots of NCO/CF-3//CC compared with other similar systems.

To explore the electrochemical performance of NCO/CF-3, a two-electrode battery-supercapacitor hybrid system (BSH) based on NCO/CF-3 and carbon cloth (CC), NCO/CF-3//CC, was evaluated with 2 M KOH as the electrolyte. Fig. 6a and b show the CV and GCD curves of NCO/CF-3//CC, respectively. Obviously, a 1.6 V of stable potential window can be determined without observable polarization. The CV curves retain the quasi-rectangular shape without obvious distortion even at a scan rate of 50 mV s^−1^ with a high rate capability. According to the GCD curves, the calculated specific areal capacity of this hybrid system is 6.2, 5.8, 5.5, 4.3, 3.6, 2.9, 2.0 C cm^−2^ at a current density of 3, 5, 8, 10, 20, 30, 50 mA cm^−2^, respectively. The energy density and power density of BSH were calculated and illustrated in Table S1† and Ragone plots in Fig. 6c. When the power density is 2.4 mW cm^−2^, the energy density is 1.38 mW h cm^−2^. It shows comparable energy/power performance to those of most previously reported similar systems in Fig. 6c such as Ni(OH)_2_–Cu/Ni foam//rGO, Ni–Cu hydroxide/carbon fibre paper//rGO, Cu_0.2_Ni_0.8_O//rGO, and Ni–Cu oxide//rGO, thus highlighting its superior energy storage performance.^21,44–46^

### 2.5 The application performance of flexible solid-state energy storage system f-NCO/CF-3//CC

**Fig. 7:**
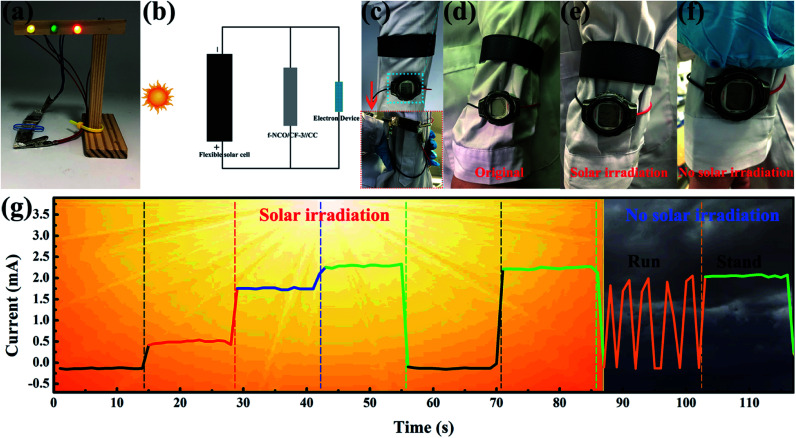
(a) The optical picture of two f-NCO/CF-3//CC connected in series lighting up 3 LEDs in parallel. (b) The schematic diagram of a self-powered and working system. (c) The optical picture of the self-powered system equipped on a lab coat and the inset shows f-NCO/CF-3//CC is designed into the strap of the watch. (d–f) The optical pictures of the electronic watch display under original, solar irradiation and no solar irradiation states. (g) The current response curves of the pressure sensor in the of the self-powered system based on f-NCO/CF-3//CC.

Finally, to certify the practical application availability of NCO/CF-3 as the flexible energy storage electrode material in future wearable electronics, several types of application were tested as shown in Fig. 7. Firstly, the flexible solid-state BSH was assembled based on the NCO/CF-3 electrode and CC electrode with KOH/PVA as the gel electrolyte, called f-NCO/CF-3//CC. As shown in Fig. 7a, two f-NCO/CF-3//CC devices connected in series can light up yellow, green and red light-emitting diodes (LED) in parallel, which increases the total voltage to 3.2 V. Furthermore, a self-powered and working system was constructed, connected as schematic in Fig. 7b, where f-NCO/CF-3//CC was connected with a flexible solar cell and an electronic device such as an electronic watch or a pressure sensor in parallel, respectively. When the solar cell is exposed to sunlight, it charges f-NCO/CF-3//CC and simultaneously provides energy for electronic devices. Then, when in a dark environment, f-NCO/CF-3//CC can act as backup power for electronic devices. As shown in the inset of Fig. 7c, when f-NCO/CF-3//CC is connected with an electronic watch, it can be designed into the strap of an electronic watch. As shown in Fig. 7d, at the beginning of the solar cell exposed to sunlight, there is no display on the watch. After several minutes of solar irradiation, the electronic watch shows the display (Fig. 7e), and the f-NCO/CF-3//CC is charged by the solar cell. Therefore, although the solar cell is completely covered under an environment without solar irradiation, the watch still displays the “time” supplied by the backup power, *i.e.*, the charged f-NCO/CF-3//CC, as shown in Fig. 7f. Moreover, in the self-powered pressure sensor working system, the pressure sensor is under the wearer's foot. The pressure sensor shows different current response under light when the wearer makes different postures such as lifting a leg, tapping the ground lightly, stepping on the ground, and stand upright, as shown in Fig. 7g. Note that the harder the foot presses, the greater the current response. When there is little solar irradiation, the pressure sensor can still be successfully powered, as shown in the last two stages in Fig. 7g. The f-NCO/CF-3//CC as the backup supply can successfully power the pressure sensor, which displays the current response no different from that under sunlight. These results show that flexible NCO/CF-3 is promising to be applied in energy storage devices to power various portable and wearable devices in the future.

## Conclusions

In this work, the NCO/CF electrode with excellent conductivity and electrochemical performance was prepared using a two-step electroless deposition and electrochemical oxidation method, which is promising for large-scale production. The flexible NCO/CF presented not only excellent mechanical strength and toughness but also high specific capacity, 4.7 C cm^−2^, at a current density of 2 mA cm^−2^. By matching the as-prepared NCO/CF electrode with a carbon cloth, the BSH demonstrated a power density of 2.4 mW cm^−2^ when the energy density is 1.38 mW h cm^−2^. Furthermore, the flexible solid-state energy storage system based on NCO/CF electrode with a carbon cloth was assembled with gel electrolyte. In a self-powered system, it exhibited excellent wearable practical applications. These results demonstrated that the NCO/CF in this work was promising as an energy storage electrode for wearable and smart electronics in future.

## Conflicts of interest

The authors declare that they have no known competing financial interests or personal relationships that could have appeared to influence the work reported in this paper.

## Supplementary Material

NA-002-D0NA00210K-s001
