# Supplementary material for: Hierarchical Ni(OH)2/Cu(OH)2 interwoven nanosheets in situ grown on Ni–Cu–P alloy plated cotton fabric for flexible high-performance energy storage
Source: Nanoscale Adv. 2020 Jun 5;2(8):3358–66. doi: 10.1039/d0na00210k (PMC9417900; doi:10.1039/d0na00210k)
Supplement: NA-002-D0NA00210K-s001 [file NA-002-D0NA00210K-s001.pdf]

Electronic Supplementary Material (ESI) for Nanoscale Advances.
This journal is © The Royal Society of Chemistry 2020

Supporting Information

for

Hierarchical $Ni(OH)_2/Cu(OH)_2$ interwoven nanosheets in situ grown on Ni-Cu-P alloy plated cotton fabric for flexible high-performance energy storage

Man Zhou,[a] Zhihang Jin,[a, b] Lifang Su,[a, b] Kai Li,[a, b] Hong Zhao,[a,c] Jinguang Hu,[c] Zaisheng Cai,[a] and Yaping Zhao*[a,b]

[c.] *Key Laboratory of Science and Technology of Eco-Textiles, Ministry of Education, College of Chemistry, Chemical Engineering and Biotechnology, Donghua University, Shanghai 201620, P.R. China*

**E-mail addresses: zhaoyping@dhu.edu.cn (Prof. Yaping Zhao)*

[b.] *Fundamental Experimental Chemistry Center, Donghua University, Shanghai 201620, P. R. China*

c. Department of Chemical & Petroleum Engineering, Schulich School of Engineering, University of Calgary, Calgary AB T2N 4V8, Canada

EXPERIMENTAL DETAILS

1. Materials

NaOH, $NaBH_4$, $NiSO_4·6H_2O$, $CuSO_4·6H_2O$, $NaH_2PO_2·H_2O$, $Na_3C_6H_5O_7·2H_2O$, KOH, and PVA etc, all the reagents were of analytical grade, purchased from Sinopharm Chemical Reagent Co., Ltd and used as received without further purification.

2. Synthesis of Ni/Cu/CFs

Ni/Cu/CFs was prepared by a simple electroless plating process. The commercial cotton fabric (CF) was immerged into 10 g/L NaOH solution for 2 h at a temperature of 85 ^oC. Then

it was taken out, washed by distilled water and dried at 60 °C overnight. After these treatments, the clean CF was gotten. Then, a 5 cm*5 cm of clean CF was immersed in the mixture of 2 g/L $NaBH_4$ and 0.04 g/L NaOH solution. And the solution was sonicated for 30 min, and then dried at room temperature. After that, the dried sample was placed in an electroless nickel plating bath with a pH of 10 and a temperature of 80°C for 2h. The composition of the electroless plating solution included 40 g/L $NaH_2PO_2·H_2O$, 50 g/L $Na_3C_6H_5O_7·2H_2O$, 30 g/L $NiSO_4·6H_2O$ and 2.5, 3.3, or 5 g/L $CuSO_4·6H_2O$. Finally, the plated samples were rinsed thoroughly with distilled water and dried at 60 °C overnight, named Ni/Cu/CF-1, Ni/Cu/CF-2 and Ni/Cu/CF-3 respectively, after different concentrations of $CuSO_4·6H_2O$. After electroless plating process, a uniform Ni-Cu-P layer with the thickness was coated on the CF. For the purpose of comparison, Ni/CF was prepared without $CuSO_4·6H_2O$ in the electroless plating bath.

3. Synthesis of NCO/CFs

The electrochemical oxidation was conducted in a three-electrode configuration with a platinum counter electrode, a Hg/HgO electrode as the reference electrode, and 2 M KOH as the electrolyte. The oxidation of the Ni/Cu/CFs (1 cm*1.5 cm) was carried out in a potential window of -0.9~1V at a scan rate of 10 mV s^{-1} for 100 cycles of the cyclic voltammetry. NCO/CF-1, NCO/CF-2, and NCO/CF-3 (NCO/CFs) were prepared based on Ni/Cu/CF-1, Ni/Cu/CF-2 and Ni/Cu/CF-3. Compared to the original CF (1 cm*1.5 cm), the mass loading of NCO/CF-1, NCO/CF-2 and NCO/CF-3 is around 0.0340, 0.0387 and 0.0452g.

4. Characterizations

The corresponding EDS mapping images were obtained under a field emission scanning electron microscope (FESEM, S-4800, HITACHI, Japan). The morphologies were observed using a field emission scanning electron microscope (FESEM, S-4800, HITACHI, Japan). The X-ray diffraction (XRD) patterns of film electrodes were recorded using a Rigaku D/Max 2550 X-ray diffractometer with Cu Ka radiation at 40kV and 300mA. The X-ray

photoelectron spectroscopy (XPS) analysis of film electrodes was performed by a PHI 5000C X-ray physical electronics photoelectron spectrometer with Mg Ka radiation at 15kV and 500W. A Micromeritics TriStarII 3020 surface area and porosity analyzer was utilized to study the pore structure of the samples.

5. Electrochemical Measurements

Electrochemical measurements of the electrode materials were carried out at room temperature in a standard three-electrode configuration on a CHI 760D (Chenhua, Shanghai) workstation with 2M KOH aqueous solution as the electrolyte. NCO/CF electrode (1cm*2cm) was used as the working electrode. A platinum electrode and a a Hg/HgO electrode were used as counter and the reference electrode, respectively. The specific capacity (C, C cm^{-2}) of NCO/CF was calculated by the Equation (1) as follows:

$$C = \frac{I \cdot \Delta t}{S} \qquad (1)$$

Where I (A) is the discharge current. Δt (s) is the discharge time. S (cm^2) is the geometric area of the working electrode.

Electrochemical measurements of the battery-supercapacitor hybrid system, NCO/CF-3//CC, were carried out in the two-electrode configuration, also on a CHI 760D (Chenhua, Shanghai) workstation with 2M KOH aqueous solution as the electrolyte. NCO/CF electrode (1cm*2cm) and the carbon cloth (CC) (1cm*2cm) were two electrodes. Energy density (E, mWh cm^{-2}) and power density (P, mW cm^{-2}) from the charge/discharge curves can be calculated by the Equation (2) and (3) as follows:

$$E = \frac{1}{7.2} C \cdot \Delta V \qquad (2)$$

$$P = 3.6 \times 10^{6} \times \frac{E}{\Delta t} \qquad (3)$$

Where ΔV is the potential window (V).

Further, the flexible solid-state energy storage f-NCO/CF//CC was assembled based on NCO/CF as the positive electrode (1 cm *1 cm) and the CC (1 cm *1 cm) as the negative electrode with KOH/PVA gel electrolyte. The KOH/PVA gel electrolyte was prepared by mixing 2.5 g PVA and 1.6 g KOH into 30.0 mL distilled water and stirring at 85 ℃, until the solution became transparent. The cellulose separator was sandwiched in between two electrodes, subsequently sealed by the clip for further use.

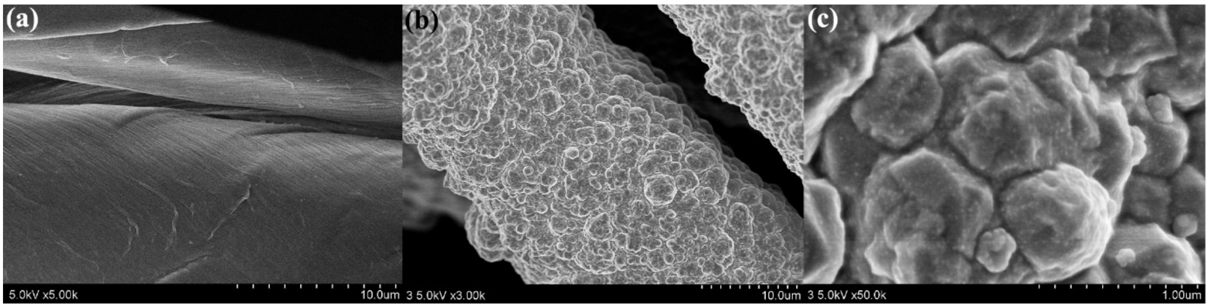

Figure S1. FESEM images of cotton fabric (a) and Ni/Cu/CF-3 (b and c).

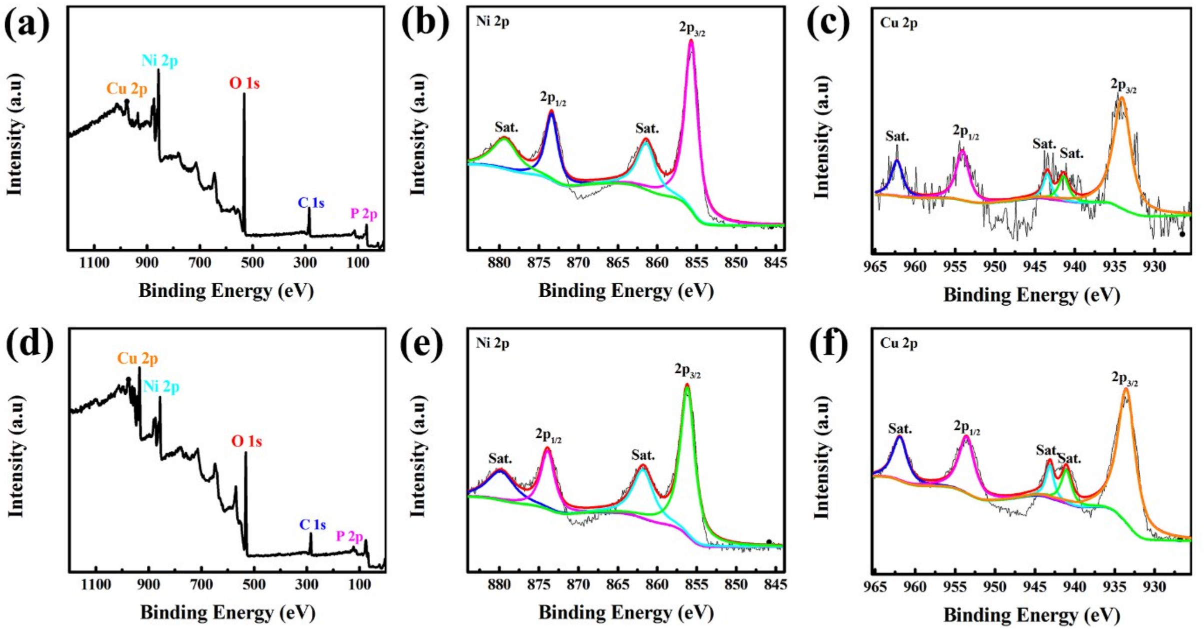

Figure S2. (a) XPS survey spectrum, and high-resolution XPS spectra for (b) Ni 2p, and (c) Cu 2p of NCO/CF-1. (d) XPS survey spectrum, and high-resolution XPS spectra for (e) Ni 2p, and (f) Cu 2p of NCO/CF-2.

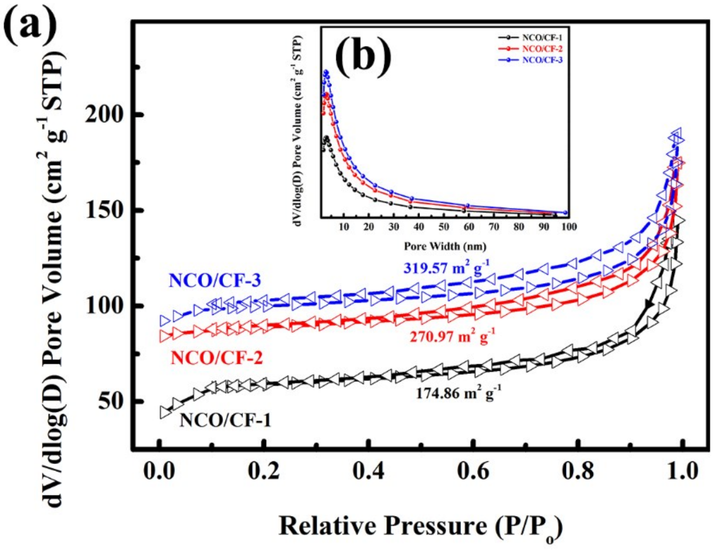

Figure S3. (a) N_2 adsorption-desorption isotherms and (b) pore size distribution based on BJH method of NCO/CFs.

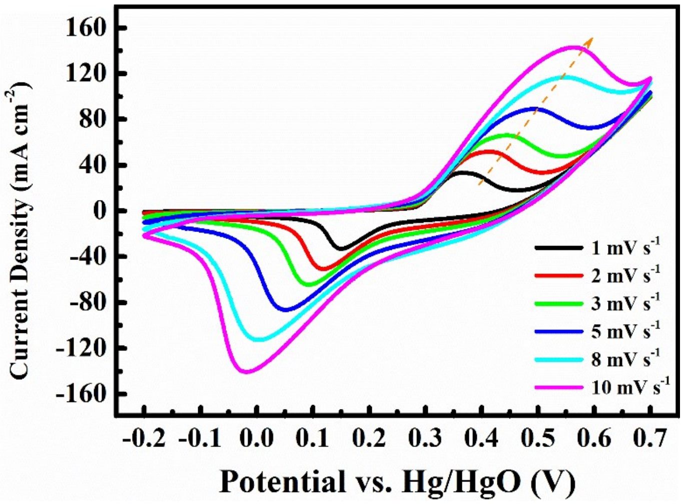

Figure S4. CV curves for NCO/CF-3 at various scan rates.

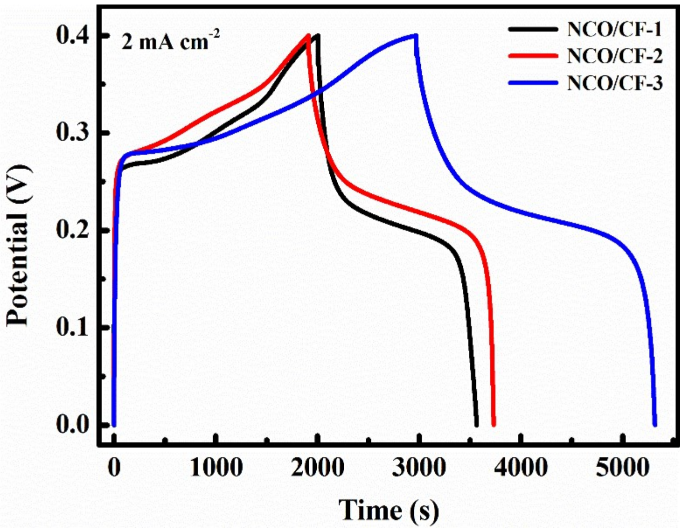

Figure S5. GCD curves of NCO/CFs at a scan rate of 2 mA cm^{-2}.

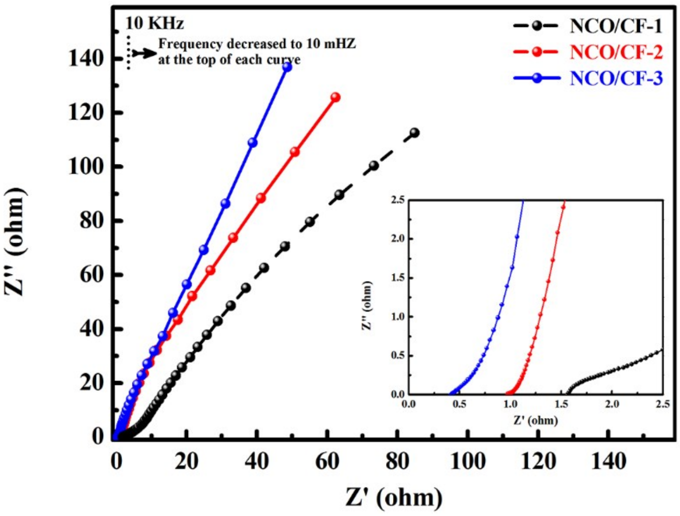

Figure S6. EIS curves of NCO/CFs.

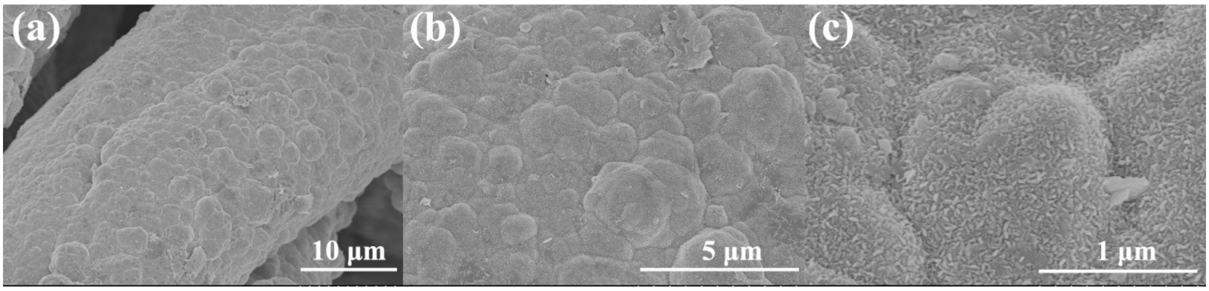

Figure S7. FESEM images of NCO/CF-3 with different magnifications, 3k (a) ,10k(b) and 50k(c), after 5000 GCD cycles.

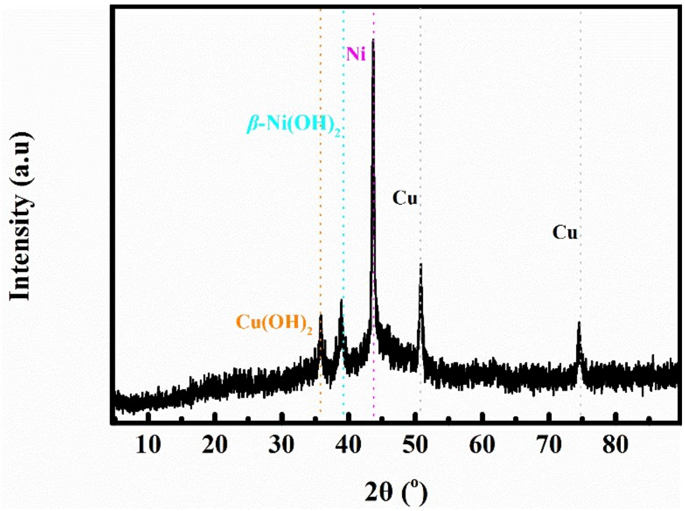

Figure S8. XRD pattern of NCO/CF-3 after 5000 GCD cycles.

Table S1. The electrochemical performance for the NCO/CF-3//CC BSH

**Current Density ($mA\ cm^{-2}$)**	**Specific Capacity ($C\ cm^{-2}$)**	**Energy Density ($mW\ h\ cm^{-2}$)**	**Power Density ($mW\ cm^{-2}$)**
3	6.2	1.38	2.4
5	5.8	1.29	4.0
8	5.5	1.22	6.4
10	4.3	0.97	8.0
20	3.6	0.80	16.0
30	2.9	0.64	24.2
50	2.0	0.44	40.1
